# Electrophysiological Correlates of Amplified Emotion-Related Cognitive Processing Evoked by Self-Administered Disgust Images

**DOI:** 10.3390/brainsci14060525

**Published:** 2024-05-21

**Authors:** Valentina Bianco, Annalisa Bello, Rocco Luca Cimmino, Giuliana Lucci, Elena Mussini, Rinaldo Livio Perri, Renato Borgatti, Francesco Di Russo

**Affiliations:** 1 Department of Brain and Behavioral Sciences, University of Pavia, 27100 Pavia, Italy; 2 Department of Movement, Human and Health Sciences, University of Rome “Foro Italico”, 00135 Rome, Italy; elena.mussini@outlook.com (E.M.); francesco.dirusso@uniroma4.it (F.D.R.); 3 Department of Mental Health, “Psychiatric Service Diagnostic and Care (Impatient Unit for Acute Patients)”, ASL Lecce (The Local Health Authority), 73100 Lecce, Italy; annalisabello@gmail.com; 4 Cognitive Psychology Association (APC), 73100 Lecce, Italy; 5 Dipartimento di Scienze Umane, Facoltà Scienze della Formazione, Università degli Studi “G. Marconi”, 00193 Rome, Italy; g.lucci@unimarconi.it; 6 Department of Psychology, University Niccolo Cusano, 00166 Rome, Italy; rinaldo.perri@unicusano.it; 7 Child Neurology and Psychiatry Unit, IRCCS Mondino Foundation, 27100 Pavia, Italy; renato.borgatti@mondino.it; 8 IRCCS Santa Lucia Foundation, 00179 Rome, Italy

**Keywords:** event-related potentials (ERPs), late posterior positivity (LPP), emotion processing, affective chronometry, disgust

## Abstract

In the processing of emotions, the brain prepares and reacts in distinctive manners depending upon the negative or positive nuance of the emotion elicitors. Previous investigations showed that negative elicitors generally evoke more intense neural activities than positive and neutral ones, as reflected in the augmented amplitude of all sub-components of the event-related potentials (ERP) late posterior positivity (LPP) complex, while less is known about the emotion of disgust. The present study aimed to examine whether the LPP complex during the processing of disgust stimuli showed greater amplitude than other emotion elicitors with negative or positive valences, thus confirming it as a neural marker of disgust-related negativity bias at earlier or later stages. Thus, in the present study, we leveraged the ERP technique during the execution of an affective self-administered visual stimuli task to disentangle the neural contributions associated with images of positive, negative, disgust, or neutral emotions. Crucially, we showed that handling with disgust elicitors prompted the greatest neural activity and the highest delay during self-administration. Overall, we demonstrated progressive neural activities associated with the unpleasantness of the emotion elicitors and peculiar processing for disgust compared with all other emotions.

## 1. Introduction

The environment is unpredictable, and the quick evaluation of events is necessary for fight/flight responses. Based on an evolutionary perspective, some environmental stimuli have salient meanings, requiring very fast processing (see [1] for review). Accordingly, adaptive success depends on the efficiency in detecting and responding to emotional stimuli with high survival significance as in the case of food stimuli, mating partners, or signals of threat (e.g., [2,3]). Emotions are complex phenomena primarily concerned with the evaluation of the elicitors capturing our attention and with the immediate action selection among several alternatives [4]. There are two models for classifying emotions. The categorical model uses six basic emotion classes such as anger, disgust, fear, joy, sadness, and surprise, or expressive classes such as boredom and confusion. The advantage of this model is that it represents human emotions automatically with easy-to-understand labels. The dimensional model classifies emotion using two main parameters such as valence and arousal. The valence defines the positivity or negativity of emotion and ranges from unpleasant feelings to happiness. The arousal denotes the excitement level that the emotion represents, and it ranges from boredom to high excitement. The advantage of this model is that it can capture fine emotion concepts that differ only to a small extent as compared with broad emotion categories (e.g., [5]). These models can be integrated to identify an emotion and to rate it finely. According to the categorical model, one category is disgust, which represents a feeling of aversion towards potential sources of illness, disease, and contamination [6]. Disgust processing has robust consequences on (a) physiological responses [7], stimulating the sympathetic and parasympathetic nervous systems [8]; and (b) cognition and behavior [9], having a slower and longer-lasting onset and affecting ongoing processing, in that disgust-specific attention bias occurred even in the absence of voluntary attention [10,11]. A widely used method to assess emotions is the subjective rating of the International Affective Picture System (IAPS, [12]). Based on the dimensional model, in the IAPS, visual images are rated based on valence (from pleasant to unpleasant) and level of perceived arousal (from calm to exciting).

A plethora of neurophysiological studies, using the IAPS, demonstrated that events eliciting an emotional reaction have facilitated perceptual processing (e.g., [13]) and increased attentional allocation [14,15]. Neuroimaging evidence highlights the involvement of many cerebral regions in the processing of affective stimuli (e.g., [16,17]), including the amygdala, the insula, medial prefrontal, anterior cingulate, premotor, temporal, and occipital cortices. Nevertheless, although brain-imaging methods provide a fine spatial resolution, event-related potentials (ERPs) are more suitable for investigating the time course of brain activities during the processing of emotional events. 

Many studies have investigated how emotional valence impacts on ERPs in healthy individuals (for a review, [18,19]) and it has been found that several ERP components are increased by emotional compared with neutral stimuli. Emotional valence affects both early ERP components, reflecting automatic attentional responses [20,21]. However, since emotion regulation typically takes time to enact, later ERP components, including the late positive potential (LPP, [18]), are those more strongly associated with emotional processing. The LPP is a complex of positive-going deflections typically starting at approximately 300 ms post-stimulus onset and persisting for a long time (e.g., [15]), even for six seconds [22]. The LPP exhibits distinct peaks of amplitude, typically larger for stimuli with positive or negative valence compared with neutral ones, irrespective of stimulus rarity or task relevance (e.g., [23,24,25,26]. While earlier peaks of LPP appear to be primarily sensitive to arousal level [23,27], the later peaks seem associated with emotional valence (e.g., [13,28,29,30,31]). The earliest LPP rises in parietal–occipital areas at approximately 300–400 ms and has been interpreted as an emotional response to highly arousing stimuli. Subsequent stages show a progressive anteriorization towards parietal, frontal, and prefrontal brain areas, suggesting enhanced and sustained attention to emotional stimuli due to sensory–motor integration and motivational areas (e.g., [31,32,33,34,35]). The increase in parietal LPP activity has been associated with subjective emotional arousal (e.g., [36]), while, in more anterior areas, it has been associated with a greater need for resources for cognitive processing [37]. These studies further point to strong bi-directional influences between parietal–occipital and frontal cortices, leading to bottom-up and top-down processes interacting for the processing of emotional stimuli. Overall, given the evidence that more arousing stimuli increase the LPP, its amplitude can be considered as an index of emotional response intensity (see [38] for review).

While a large body of ERP research in emotion investigated threat or fear [39], comparatively less attention has been given to disgust. Jin and co-authors [40] investigated how the duration of exposure to disgust vs. neutral images affected the performance of a visual search task and showed a unique neural processing mechanism for the former. Liu and co-authors [41] compared disgust and angry stimuli during a dot-probe task and demonstrated reversed patterns of cortical activities during early and late processing stages. Together with a subsequent study [42] using the same task, but introducing the emotion of fear, all these investigations suggest a crucial impact of dealing with disgust stimuli on attentional mechanisms.

Focusing on the late processing of disgust stimuli, ERP studies have observed larger LPP for disgust compared with neutral and fear primes [43,44], as well as larger early LPP peaks for disgust compared with sadness (470–550), with a reversed effect for the later interval (880–1000 ms) [45]). Wang and colleagues [45] proposed an explanation rooted in evolution: a disgusting signal requires quick and immediate action to ensure survival, whereas a slower but longer-lasting response may be more evolutionarily advantageous in dealing with negative events such as sadness [45,46]. Briesemeister and co-authors [47] found that processing disgust-related words showed a disadvantage compared with neutral ones, a pattern not observed for other negative emotions. Further, other ERP studies have reported an increased amplitude associated with disgust during the LPP period, proposing that disgust may attract sustained attention, thereby enhancing memory processing. However, these studies did not directly compare disgust with other negative emotions (e.g., [45,48,49,50]), i.e., disgust was not considered as a separate category. Further, the effect of disgust was evident in both the parietal and frontal LPP at approximately 400 and 700 ms, respectively [50].

Last, it is important to highlight the significance of better understanding “affective chronometry” [51], which refers to the temporal dynamics of emotional responses. This understanding is crucial for linking the subjective experience of emotion with the objective neural measures associated with the affective process. Interestingly, Perri and co-authors [31] employed a motor task in which the participants freely decided to self-administer positive, negative, or neutral stimuli, thus creating their own emotional experience. Furthermore, there is evidence that foreknowledge of the emotional valence of an upcoming stimulus facilitates emotion regulation and enhances the LPP [31,52,53].

To better understand the connection between emotion and cognition, the present study utilized the ERP technique to investigate the impact of exposure to positive and negative images on both behavioral performance and neural activity. However, we treated disgusting stimuli as a distinct category. Crucially, differently from previous investigations, the present study combined all ‘other’ negative emotions, contrasting them to disgust, and included positive emotions to explore the comparison with stimuli eliciting emotions of opposite valence. To generate a strong conscious expectancy regarding the positive or negative nature of the images and to elicit robust emotion-related LPP, participants in this study were informed in advance about the emotional valence of the upcoming stimulus and were responsible for self-administrating the stimulus themselves.

The aim of the present study was to compare the LPP complex associated with the processing of the disgusting stimuli with respect to other emotions. Differently from previous investigations focusing on the temporal processing of disgust stimuli in relation to attentional mechanisms (e.g., [40,41,42]), here we prioritize the effect of “getting ready” to emotionality on the subsequent processing of disgust stimuli. We hypothesize that if disgust elicits the most unpleasant emotional response, this will lead to a delay in the decision to attend to this category, resulting in longer response times. Furthermore, if disgust evokes the strongest brain activity in earlier or later stages of emotional processing, we expected to observe the largest amplitude as neural markers of disgust-related processing in earlier or later stages of the LPP.

## 2. Methods

### 2.1. Participants

The sample size was determined a priori by power analysis, conducted using the G*Power 3.1.9.7 [54] which indicated the requirement of 18 participants for an effect size of f = 0.306, a power (1-β error probability) of 0.85, a correlation among repeated measures of 0.5, and an alpha probability of 0.05 for within-factors repeated measures ANOVA. These values predicted an actual power of 0.855. The effect size (f value) was calculated based on the average partial eta squared values of emotional comparisons from a previous study investigating the LPP using the same number of IAPS images (60 per condition) and similar arousal and valence levels as in the present study [55]. Therefore, 18 volunteers participated in the study (nine females; mean age = 30.5 years; SD = ±3.41). All participants were right-handed [56] and without any psychiatric, neurological, or chronic diseases. Following an explanation of the procedures, written informed consent was obtained from participants before the beginning of the experimental session. The study was approved by the University of Foro Italico Ethics Committee.

### 2.2. Stimuli and Tasks

The stimuli consisted of 240 images, comprising 180 affective images equally classified into three emotional categories: 60 positive, 60 negative, and 60 disgusting, selected from the International Affective Picture System (IAPS, [12]) and 60 scrambled images. All the emotional images were rated in the IAPS. The positive images included representations of joy, baby animals, loving families, and children playing, with a mean valence and arousal rate of 7.4 ± 0.5 and 4.5 ± 0.7, respectively. The negative pictures included representations of sadness, anger, fear, war, and disasters, with a mean valence and arousal rate of 2.8 ± 0.8 and 5.9 ±0.8, respectively. The disgust pictures were part of the negative pictures but included only those labeled as disgust, such as scenes of dirty toilets, mutilations, and spoiled food. Scenes including blood were not included since they can evoke fear. The mean valence and arousal rate of disgusting images was 2.6 ± 0.8 and 5.7 ± 0.9, respectively. Statistical analyses showed that the valence of negative and disgust images was comparable (t_(118)_ = 1.3, *p* = 0.205). The arousal levels of the three categories differed (F_(2,118)_ = 51.0, *p* < 0.001). Bonferroni’s post-hoc comparisons showed that the arousal of positive images was lower (*p* < 0.001) than the negative and disgust ones, which did not differ from each other (*p* = 0.256). Table 1 shows the list of the used images. The scrambled pictures were built by scrambling 20 negative, 20 positive, and 20 disgust pictures, randomly selected from each emotional category using graphic software. Scramble pictures served as control stimuli, as they retained the perceptual features of original images while removing their affective content (e.g., [57,58]). In summary, four categories were used in the experiment: disgust (D), negative (N), positive (P), and scrambled (S).

During the experiment, participants were comfortably seated in front of a computer monitor placed at a distance of 120 cm from their eyes. Visual stimuli were displayed against a black background and were administered using Presentation Software 24.0 (Neurobehavioral System, Inc.; Albany, CA, USA). A fixation point—represented by a yellow circle subtending 0.15° × 0.15° of the visual angle—remained steady in the center of the computer monitor throughout the entire experiment.

**Figure 1:**
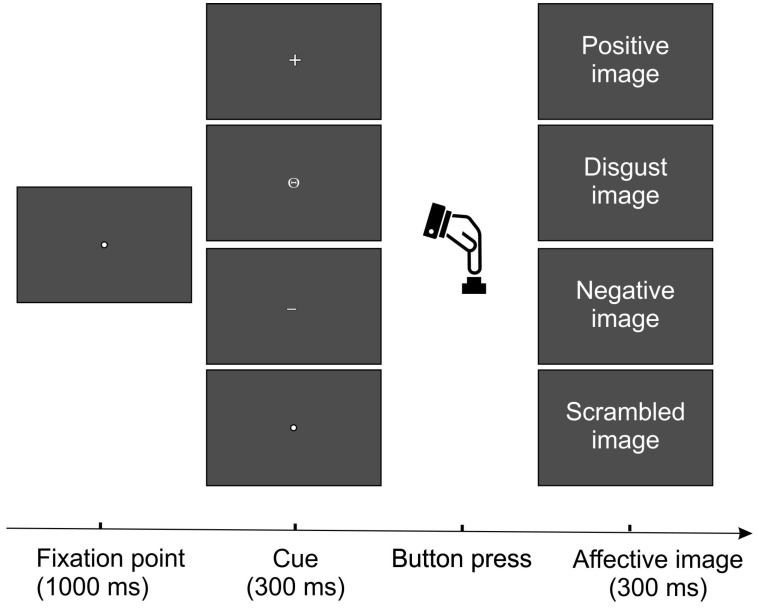
Representation of the task procedure.

The experimental task was adapted from Perri and co-authors [31] since they showed that self-administrated emotional pictures may enhance arousal level and the response to valence and therefore may evoke strong LPP activity. As shown in Figure 1, during the task, participants were asked to fix a point that remained in the center of the screen for 1000 ms and to press a button with their right index finger after the presentation of small symbols (0.3° × 0.3°) that replaced the fixation point for 300 ms. No time restrictions were given for the button press. According to the symbol type, pressing the button triggered the appearance of an emotional picture on the screen, visible for 300 ms. Participants were informed in advance about the association between the symbol type and the emotional category, as follows: positive stimuli were preceded by a cross (+); disgusting stimuli by a circle with a horizontal dash inside (Θ); negative stimuli by a dash (−); and scrambled by a dot (∙). The entire task was divided into two blocks, randomly presented, and counterbalanced across participants. Each block comprised 120 pictures, equally divided across each category (i.e., 30 pictures per category). The duration of each block changed depending on the subjective response time; anyway, the mean duration was approximately 20 min for a block.

### 2.3. Electrophysiological Recording and ERP Analysis

The EEG was measured from 64 electrodes mounted according to the 10-10 International System using the BrainVision^TM^ system (BrainProducts GmbH, Munich, Germany). The left mastoid was used as reference. Horizontal and vertical eye movements were separately measured with bipolar electrodes. All electrode impedances were maintained below 5 KΩ. The EEG was digitized at 250 Hz, amplified (band pass of 0.01–80 Hz including a 50 Hz notch filter), and stored for offline averaging. Eye movements were attenuated using the Gratton and Coles algorithm [59], which is the equivalent of most complex methods, such as independent component analysis (e.g., [60]). To reject epochs contaminated by sources of signal noise, artifact rejection procedures were applied as follows: first an amplitude threshold of ±120 μV was applied. Then, following the automatic procedure, trials that were still contaminated by artifacts like ocular movements and muscular contractions were manually discarded. Last, an average of 89% of trials was retained. The four emotional categories (D, N, P, S) were separately averaged into non-overlapping epochs of 1600 ms epochs (from 100 ms before to 1500 ms after the stimulus onset). The baseline was selected as the mean voltage over the beginning 100 ms of the averaged epochs. To further reduce high-frequency noise, the group-averaged ERPs were loss-pass filtered (i.e., Butterworth) at 40 Hz.

**Figure 2:**
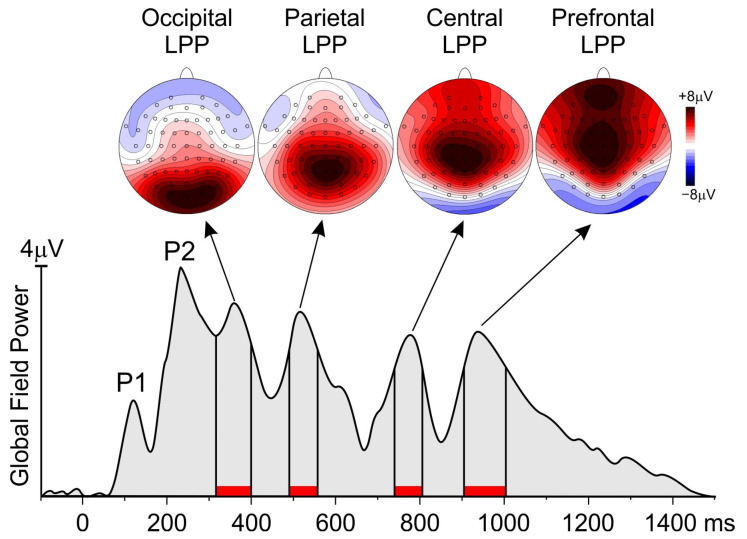
Global field power of the four conditions averaged together showing the intervals (red bars) included in the analysis. The topographical maps show the scalp distribution of the late positive potentials (LPP) in those intervals.

The “collapsed localizer” method [61] was used to identify the electrodes to include in statistical analysis. Relatedly, to identify the interval of analysis, the global field power (GFP) was calculated. The GFP is a reference-free method that reduces all the scalp channels to a single wave corresponding to a global measure of scalp potential field strength. It is calculated as the root summed square of the voltage of all recording electrodes simultaneously at each time point (e.g., [62]). More specifically, the GFP was obtained from the grand-averaged waveforms across all the participants and conditions. The GFP maxima identify the peaks of the more relevant ERP components. As shown in Figure 2, the GPF showed six peaks at 120, 230, 360, 516, 776, and 936 ms. While the first two peaks represent the P1 and the P2 components associated with early visual processing, the other four later positive peaks were included in the analysis. The analysis interval was defined as the period where the GPF was at least 80% of its peak value. This GFP approach selected the following intervals: 316–400 ms, 490–556 ms, 740–806 ms, and 904–1004 ms, in which the mean amplitude was calculated for statistical purposes in each emotional condition. The electrodes with an amplitude larger than 80% of the maximum value in the intervals selected by the collapsed localizer were jointed in spatial pools and considered for statistical analysis. As shown by the scalp topographies in Figure 1, the first interval had a medial occipital focus and was measured as a pool containing PO3, POz, PO4, O1, Oz, and O2, and was defined as the occipital pool. The second interval had a medial parietal focus and was measured as a pool containing CP1, CPz, CP2, P1, Pz, and P2 and was defined as the parietal pool. The third interval had a medial central focus and was measured as a pool containing C1, Cz, C2, CP1, CPz, and CP2 and was defined as the central pool. The fourth interval had a medial prefrontal focus and was measured as a pool containing Fp1, Fp2, AF1, AFz, and AF2 and was defined as the prefrontal pool.

### 2.4. Statistical Analysis

First, Wilk–Shapiro and Levene tests for normal distribution and equality of variance, respectively, were performed, showing no violation of the distribution and of the sample homoscedasticity (*p* < 0.05). Response time was submitted to one-way analysis of variance (ANOVA) with the picture emotional valence (D, N, P, S) as a within-subjects factor. Likewise, ERP data for each interval and pool of electrodes were submitted to one-way ANOVA with the emotional valence (D, N, P, S) as a within-subjects factor. To evaluate the effect size of the results, the partial eta squared (η_p_^2^) was reported. Post-hoc comparisons were conducted using the Bonferroni method. The alpha level was fixed at 0.05. All statistical analyses were executed using the Statistica 12.0 software (StatSoft Inc., Tulsa, OK, USA).

## 3. Results

### 3.1. Behavioral Data

The RT ranged from 1850 to 3670 ms. The ANOVA conducted on the response time (RT) showed a significant main effect of valence (F_3,51_ = 5.5; *p* = 0.002, η_p_^2^ = 0.232). Post-hoc comparisons revealed that the RT relative to cues for disgust stimuli (2909 ms ± 584) was significantly longer compared with the other three emotional valences (negative: 2733 ms ± 512, *p* = 0.034; positive: 2729 ms ± 501, *p* = 0.031; scrambled: 2635 ± 527, *p* < 0.001), which did not differ each other.

### 3.2. ERP Data

**Figure 3:**
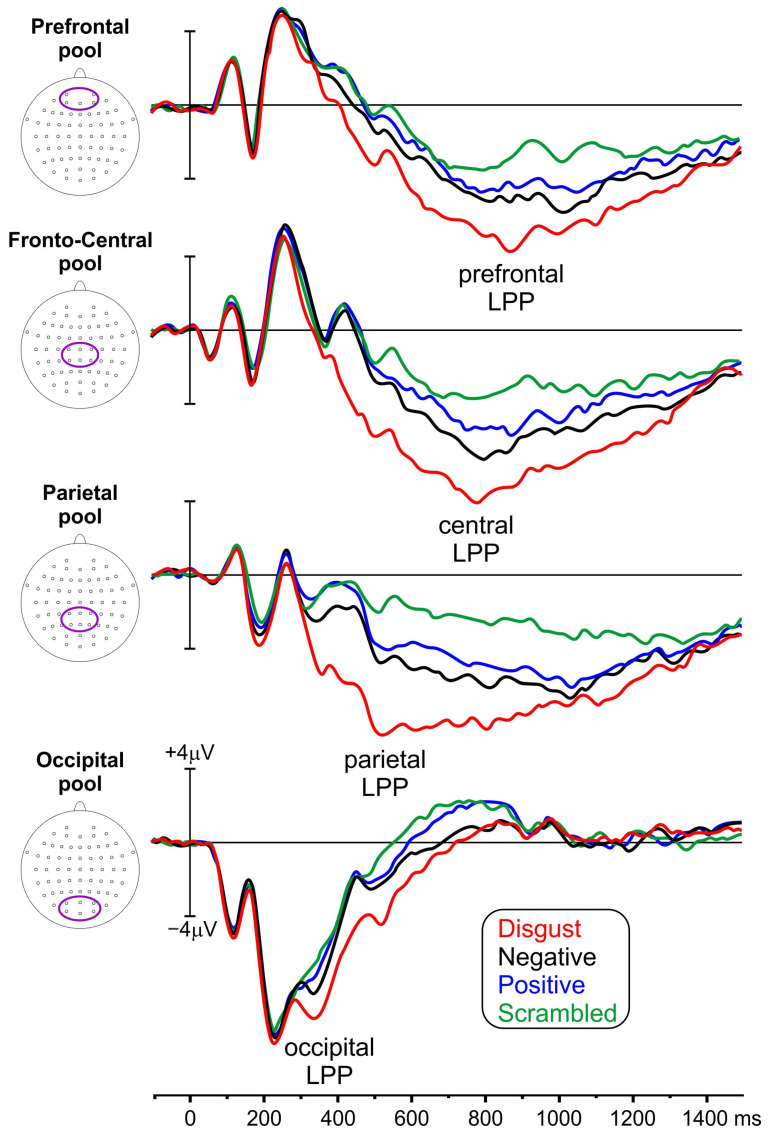
ERP waveforms from the four conditions (indicated by distinct colors) presented at the prefrontal, central, parietal, and occipital electrode pools. The electrodes included within each pool are shown by the oval inside the head’s representations (LPP = late positive potential).

Figure 3 shows the ERP waveforms across the four conditions in the selected pools of electrodes. Following the early P1 and the P2 visual components (which were comparable across conditions), the emotion-related LPP emerged at medial occipital areas, reaching its peak at approximately 350 ms. Subsequently, it was evident at medial parietal, central, and prefrontal areas at approximately 510 ms, 785 ms, and 880 ms, respectively. In terms of amplitude, the ERP waveforms elicited in the disgust condition exhibited larger amplitude compared with the other conditions, followed by the negative and the positive conditions.

The ANOVA conducted on the first occipital interval showed a significant main effect of valence (F_3,51_ = 4.8; *p* = 0.005, η_p_^2^ = 0.203). Post-hoc comparisons revealed that the amplitude for disgust stimuli (9.30 ± 1.18 μV) was larger than the other three conditions (negative: 7.99 ± 0.96 μV; positive: 7.07 ± 0.81 μV; scrambled: 6.76 ± 0.79 μV, all *p*s < 0.001). Moreover, the amplitude for negative was larger than for positive (*p* = 0.036) and scrambled stimuli (*p* = 0.009). However, the positive and the scrambled conditions did not differ from each other.

The ANOVA conducted on the second parietal interval showed a significant main effect of valence (F_3,51_ = 7.6; *p* < 0.001, η_p_^2^ = 0.321). Post-hoc comparisons revealed that the amplitude for disgust stimuli (8.11 ± 0.98 μV) was larger than for the other three conditions (negative: 4.73 ± 0.58 μV; positive: 3.88 ± 0.51 μV; scrambled: 1.94 ± 0.43 μV, all *p*s < 0.001). Additionally, the amplitude for negative stimuli was larger than for scrambled (*p* < 0.001). The amplitude for positive was larger than for scrambled stimuli (*p* = 0.005). The comparison between the negative and the positive conditions did not yield significant differences.

The ANOVA conducted on the third central interval showed a significant main effect of valence (F_3,51_ = 6.8; *p* < 0.001, η_p_^2^ = 0.287). Post-hoc comparisons revealed that the amplitude for disgust stimuli (9.07 ± 1.13 μV) was larger than for the other three conditions (negative: 6.62 ± 0.74 μV; positive: 5.31 ± 0.65 μV; scrambled: 3.67 ± 0.46 μV, all *p*s < 0.001). The amplitude for negative was larger than for positive (*p* = 0.033) and scrambled stimuli (*p* = 0.003). The amplitude for positive was larger than for scrambled stimuli (*p* = 0.007; see Figure 2).

**Figure 4:**
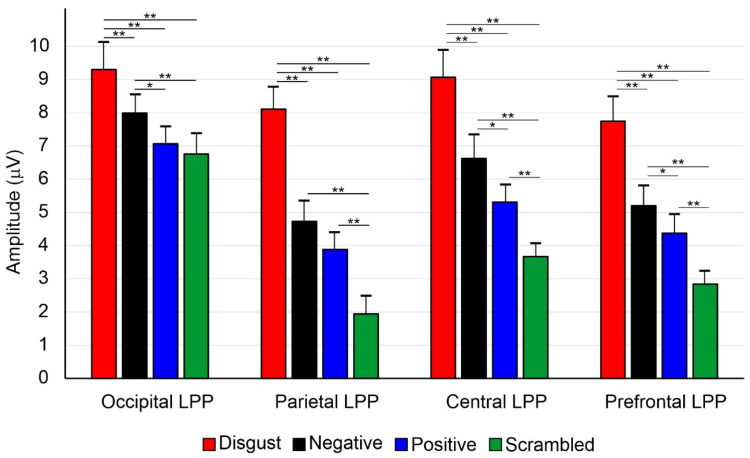
Post-hoc comparisons among the studied conditions. The vertical lines indicate the 95% confidence interval. * *p* < 0.05, ** *p* < 0.01.

The ANOVA conducted on the fourth prefrontal interval showed a significant main effect of valence (F_3,51_ = 5.3; *p* = 0.003, η_p_^2^ = 0.244). Post-hoc comparisons revealed that the amplitude for disgust (7.76 ± 1.15 μV) was larger than for the other three conditions (negative: 5.23 ± 0.1.06 μV; positive: 4.39 ± 0.88 μV; scrambled: 2.85 ± 0.59 μV, all *p*s < 0.001). The amplitude for negative was larger than for positive (*p* = 0.036) and scrambled stimuli (*p* < 0.001). The amplitude for positive was larger than for scrambled stimuli (*p* = 0.005). All amplitudes and comparisons are presented in Figure 4.

## 4. Discussion

In the present study, we have deepened our understanding of emotion processing by demonstrating that encountering disgusting stimuli triggers peculiar behavioral responses and neural activities compared not only to neutral and positive stimuli but also to other negative emotion elicitors of comparable unpleasantness (valence) and arousal level. We showed that disgusting stimuli are differentially elaborated at several points during the information processing stream, as indexed by the timing and topographical distribution of the multiple stages of the LPP complex.

First, at the behavioral level, we showed that affective cueing largely influenced the implicit delay in the self-administration of aversive views. Specifically, when the cue indicated the delivery of disgust-inducing stimuli, the time interval before participants self-administered images was longer than when the cue signaled the upcoming presentation of negative, positive, or scrambled events. Notably, the response times preceding disgust-inducing images were also longer than those preceding all other negative emotional images, suggesting distinctive processing for this specific type of unpleasant emotion. This finding is consistent with a previous study finding that during an attentional task, response times were slower for disgust targets compared with fear and neutral targets [63,64]. Crucially, Zimmer and colleagues [49] demonstrated that disgusting sound cues redirect spatial attention away from their source to an opposite location, indicating a spatial avoidance of disgust. In agreement with this, our findings suggest that, in the case of the self-administration of emotional pictures, individuals tend to delay their engagement with disgusting stimuli as much as possible compared with all other stimulus categories.

Regarding the neurophysiological findings, this study reveals a temporal unfolding in perceptual processing, with prominent activity observed for disgust stimuli across the entire LPP complex from 400 ms until 1000 ms post-stimulus. The amplitude of the earliest LPP recorded over the occipital areas was the highest for the disgust images. Additionally, we found that the LPP amplitude was greater for negative images than for positive and scrambled images, which did not differ from each other. Therefore, this component appears to be sensitive to the negative valence elicited by the observed emotional stimuli, particularly for disgust. The observed occipital LPP may be related to previous studies acknowledging a positive wave, termed early posterior negativity (EPN), which is enhanced for highly arousing emotional stimuli [38] as well as for both pleasant and unpleasant pictures compared with neutral ones [24]. However, contrary to evidence suggesting that earlier LPPs primarily reflect a perceptual reaction to more arousing images [27], our findings suggest that in a self-administered emotion task, the valence of the stimulus is considered even at this early stage of processing.

A larger amplitude for disgusting stimuli was also evident in the parietal LPP, with negative and positive images being larger than scrambled images, in line with previous studies (e.g., [50,65]), suggesting the LPP as a reliable index of subjective emotional arousal (e.g., [36]). Crucially, it has been shown that affective informative cues enhance the LPP when they anticipate emotional stimuli compared with neutral ones [66,67]. We have now added to this body of evidence information that parietal LPP is significantly influenced by dealing with disgusting images, showing the maximum peak for this specific negative category. Therefore, the parietal LPP elicited by the present task demonstrates sensitivity to arousal levels, indicating preferential processing for disgust stimuli.

Regarding the later component of the complex, namely the central LPP, the highest peak amplitude was once again observed for the disgust stimuli, followed by negative, positive, and scrambled images. This sustained wave, peaking at approximately 800 ms, appears to be sensitive to the unpleasantness of the emotional stimuli, in line with previous evidence indicating that the later phases of the LPP complex are more strongly associated with emotional valence (e.g., [13,28,29,30,31]). The observed central LPP, occurring after the occipital and parietal LPPs, clearly provides evidence of its anteriorization, as previously found [34,35], and suggests sustained attention to emotional stimuli over time.

Afterward, the fourth component of the complex, namely the prefrontal LPP, also showed the highest peak for disgust images, followed by negative, positive, and scrambled images. A previous study investigating emotion regulation strategies observed enhanced frontal LPP activity for negative compared with neutral images [50]. Further, it has been suggested that higher frontal LPP amplitude is associated with a greater allocation of cognitive resources necessary for dealing with unpleasant stimuli [37]. Accordingly, our findings suggest that dealing with disgust stimuli has the most significant impact on the observed prefrontal LPP, suggesting that a greater amount of cognitive processing is required for this specific negative emotion.

Overall, behavioral and neurophysiological findings suggest that, although emotional stimuli are generally given equivalent weight compared with neutral stimuli, preparing to deal with and subsequently attending to disgusting stimuli requires the longest preparation time and larger neural resource allocation, even compared with other negative stimuli of comparable valence and arousal.

The presence of discrepancies among the above-referenced studies could be ascribed to the variability of the experimental designs which can lead to inconsistent findings, especially in ERP research. Relatedly, previous attentional ERP studies focusing on disgust, e.g., [40], demonstrated its critical impact on attentional mechanisms compared with angry [41] and fear [42] emotions. With the present study, we advanced the understanding of the neural processing associated with disgust also in the context of auto-administrated emotional experience, showing an augmented allocation of neural resources compared with all other emotion elicitors categories.

The results of this study further have significant relevance in clinical contexts, particularly concerning the role of disgust in various clinical disorders. For instance, a strong correlation has been established between disgust sensitivity and mental disorders such as obsessive–compulsive disorders, anxiety disorders, phobias, anorexia, and depression (e.g., [67,68,69,70,71]).

The results of the present study must be considered within the context of some limitations. First, disgust is a multifaceted emotion elicited by a variety of stimuli with very heterogeneous contents and we cannot acknowledge the potential existence of distinct neural activities for distinct categories of disgust elicitors. Second, we did not collect individual measures of “affective style”, i.e., the individual consistent disposition to emotion regulation and reactivity [72,73]. This could have accounted for individual variability in the quality and intensity of dispositional mood and emotional reactions to similar elicitors. A further limitation of the present study is the absence of individual threshold measurement for each participant (i.e., disgust propensity). As a result, we were unable to distinctively differentiate between induced disgust and subjective disgust. Future studies should include disgust propensity scores as a covariate to account for potential variations in individual emotion regulation strategies. Concerning our neurophysiological findings, it must be noted that an intrinsic limitation of this study is represented by the experimental choice of treating disgust as a separate emotion category compared with other ‘random’ positive or negative. Relatedly, as the appearance of a disgust stimulus is more predictable than all other emotion-eliciting stimuli, this might have contributed to the observed amplification of the LPP.

Last, concerning the interpretation of the RT results, it is of note to point out that we cannot fully exclude the possibility that the cue signaling disgust required a longer processing time compared with the other conditions. Future studies should counterbalance the association between cues and target events across conditions and participants.

## 5. Conclusions

In conclusion, the present study demonstrated that the foreknowledge about the emotional significance of an upcoming event not only shapes behavior but also impacts the intensity of the underlying neural activities. Specifically, we observed that having to deal with disgust-inducing events led to the greatest delay in response time (i.e., for their self-administration) and elicited the greatest brain activities at several stages of cognitive processing compared with other negative, positive, or neutral events. However, it is worth noting that the neural bases of emotions still represent a matter of debate: the locationistic perspective assumes distinctive brain structures devoted to specific discrete emotions, while the psychological constructionist perspective suggests that the emotional experience emerges from the interaction between brain functional networks (see [74] for review). The present investigation does not provide unequivocal evidence in favor of one of the two approaches but suggests that dealing with disgusting images elicits distinctive ERP activity during this specific task. Overall, our data support the notion that the LPP complex in response to affective pictures is modulated by their intrinsic motivational significance and shows preferential processing when dealing with disgust stimuli.

## Figures and Tables

**Table 1:** List of the IAPS images numbers used in this study.

	Positive Images #	Negative Images #	Disgust Images #
1	1340	1019	1270
2	1440	1050	1274
3	1460	1052	1275
4	1463	1110	1280
5	1510	1120	2141
6	1610	1200	3000
7	1610	1201	3001
8	1710	1205	3010
9	1750	1220	3011
10	2010	1525	3030
11	2030	1930	3051
12	2040	1931	3060
13	2050	1932	3061
14	2057	2100	3062
15	2058	2110	3063
16	2070	2120	3064
17	2071	2205	3068
18	2080	2683	3069
19	2091	2710	3071
20	2150	2751	3080
21	2160	2800	3130
22	2165	2900	3150
23	2170	3053	3160
24	2208	3100	3168
25	2209	3102	3190
26	2216	3110	3280
27	2224	3120	3301
28	2240	3140	3400
29	2250	3170	3500
30	2260	3230	3530
31	2270	3261	7359
32	2299	3300	7360
33	2303	5971	7361
34	2310	5972	7380
35	2311	6000	9005
36	2340	6210	9006
37	2341	6211	9042
38	2344	6230	9090
39	2345	6260	9102
40	2360	6300	9253
41	2370	6313	9270
42	2387	6360	9290
43	2388	6410	9300
44	2389	6510	9301
45	2391	6540	9320
46	2395	6550	9330
47	2530	6560	9340
48	2540	6570	9341
49	2550	6571	9342
50	2630	8480	9373
51	2650	9040	9390
52	2655	9041	9405
53	2660	9252	9480
54	4609	9410	9490
55	4624	9415	9560
56	4626	9433	9561
57	4700	9440	9582
58	5830	9495	9584
59	8420	9630	9592
60	8461	9830	9800

## Data Availability

Data are available from the corresponding author upon request. The data are not publicly available due to institutional copyright policy.
